# Determining the Impact of the Opioid Crisis on a Tertiary-Care Hospital in Central New York to Identify Critical Areas of Intervention in the Local Community

**DOI:** 10.1155/2020/3956187

**Published:** 2020-03-12

**Authors:** Sanjay K. Yadava, Stephen J. Thomas, Scott Riddell, Dongliang Wang, Timothy P. Endy

**Affiliations:** Departments of Medicine, Pathology, Public Health and Microbiology and Immunology, State University of New York, Upstate Medical University, Syracuse, USA

## Abstract

**Background:**

Central New York has been afflicted by the heroin epidemic with an increase in overdose deaths involving opioids.

**Objective:**

The objective of the study was to understand the epidemiology of hospitalizations related to a diagnosis of opioid use (OU).

**Design:**

The study was designed as a retrospective analysis of hospitalized patients admitted from January 1, 2008, to December 30, 2018, using ICD-9 and 10 codes for heroin or opiate use, overdose, or poisoning. *Setting*. The study was conducted in a tertiary-care and teaching hospital located in Central New York. *Patients*. Hospitalized patients were included as study participants.

**Results:**

Opioid use-related admissions increased from .05/100 hospital admissions in 2008 to a peak of 2.9/100 in 2018, a 58-fold increase. There were 49 deaths over the 11-year period for an overall case fatality of 1.2 per 100 OU admissions. The median age for all years was 40 years (SD of 13.7 years), and admissions were largely white caucasians (67.0% of all admissions). The mean length of stay was 8.55 days (SD 12 days), with a range of 1 to 153 days. The most frequent discharge diagnosis was due to infections (15.0% of discharge diagnoses) followed by trauma (5.8% of discharge diagnoses). Methicillin-resistant *Staphylococcus aureus* was more common in patients with OU (58.1%) than in patients with non-OU (43%) (*p* < 0.0001 by chi-square with Yates' correction). Spatial analysis was performed by zip code and demonstrated regional hotspots for OU-related admissions. *Limitations*. The limitations of this study are its retrospective nature and largely numerator-based analysis. The use of ICD codes underrepresents the true burden due to underreporting and failure to code appropriately. This study focuses on patients who are hospitalized for a medical reason with a secondary diagnosis of opioid use and does not include patients who present to the emergency room with an overdose underrepresenting the true burden of the problem.

**Conclusions:**

Our results demonstrate the impact of the opioid epidemic in one tertiary-care center and the need to prepare for the costs and resources to address addiction care for this population.

## 1. Introduction

The United States (US) and Central New York (NY) are in the midst of an unprecedented epidemic of substance use disorder (SUD) specifically to heroin and other opioids. During 1999 to 2015, approximately 568,699 people died from drug overdose in the United States [1, 2]. From 2014 to 2015, there was an increase in overdose deaths of 11.4% with 52,404 deaths in 2015 [1]. The introduction of fentanyl to heroin mixtures, in particular, resulted in a deadly combination and contributed to the increasing death rate [3]. Central NY has also been afflicted by the heroin epidemic with a dramatic increase in overdose deaths involving opioids from 6/100,000 in 2010 to 14/100,000 in 2015 [4]. The US opioid epidemic highlights the racial, age, and geographic disparity of adults with SUD and deaths from overdoses. For example, in young adults aged 20–34 years, the rate of overdose deaths was 14.2 per 100,000 and double the rate among older adults aged 55–64 years (6.3/100,000) as reported in the 2017 Annual Surveillance Report of Drug-Related Risks and Outcomes [5]. In New York State during the years 2013 to 2015, there were a total of 1,440 overdose deaths [6]. Analysis of mortality rates by regions in New York demonstrated disparities in deaths related to opioid use, with clusters of high mortality in the Hudson Valley, Catskills, western Central New York, Finger Lakes, Thousand-Island Seaway regions, Richmond County, and Long Island [6]. Examining mortality rates as compared with prescription rates for opioids, the Thousand Islands-Seaway region and Central NY, in particular, had the most number of counties with higher opioid-related mortality rates but lower opioid prescription rates, which may suggest that as prescription opioids dropped, alternatives such as heroin were being used.

Overdose deaths from opioids are the tip of the iceberg of total health-associated complications from opioids and specifically from intravenous (IV) drug use. Infections related to IV drug use (IVDU) include viral infections such as human immunodeficiency virus (HIV), hepatitis C virus (HCV), and hepatitis B virus (HBV), as well as bacterial infections of the heart valve (subacute bacterial endocarditis), spinal abscess, and osteomyelitis, necrotizing fasciitis, myositis, and skin abscesses [7–10]. Many of the bacteria involved are common skin organisms including staphylococci and streptococci, although Gram-negative bacteria and fungi have also been implicated. Of particular concern is the emergence of methicillin-resistant *Staphylococcus aureus* (MRSA) as a cause of bacterial infection in drug users [11]. During 2005–2016, surveillance data from the Center for Disease Control's (CDC's) Emerging Infections Program (EIP) were analyzed and demonstrated that people who inject drugs were 16.3 times more likely to develop invasive MRSA infections than others [11]. Hospitalizations related to substance use disorder from opioids and complication of IV drug use are not reportable; thus, the true impact at the hospital level due to the opioid epidemic is not known. One study using a representative sample of US hospitalizations from opioid use and infectious complications found a significant increase in hospitalizations from 2002 to 2012 (301,707 to 520,275, respectively), with infectious complications also increasing (3,421 to 6,535, respectively) [12]. The economic cost associated with this was estimated based on the Consumer Price Index by the Bureau of Labor and Statistics and tripled, from $4,574,263,003 in 2002 to $14,850,435,892 in 2012.

Understanding the spatial epidemiology of substance use disorder either from emergency room visits or hospitalizations can be a powerful tool to identify regional hot spots to direct limited resources and educational targeting. In a study of prescriptions and overdose cases in Massachusetts by zip code, regional overdose clusters were identified in specific hot spot counties, providing an opportunity at the local level to influence public health decisions and execution of resources [13]. The number of hospitalizations from overdose and infections related to IVDU at our teaching hospital and Central NY is unknown and is the primary purpose of this study. It is imperative that we understand the epidemiology of opiate-related hospitalizations and infections to better prepare for services and empiric therapy for this population, to identify regional hot spots that can be a point source to target education and intervention, and to understand the growing rate of antibiotic resistance to implement hospital-specific infection control interventions.

## 2. Methods

### 2.1. Study Design

This was a retrospective analysis, database review of hospitalized patients admitted to university hospital from January 1, 2008, to December 30, 2018, using ICD-9 and 10 codes for any hospital admission or discharge diagnosis for heroin or opiate use, heroin or opiate overdose, poisoning by opium, poisoning by heroin, poisoning by methadone, and opioid abuse (see supplemental [Supplementary-material supplementary-material-1] for ICD-9 and ICD-10 codes used). This study focused on patients who were hospitalized for a medical reason with a secondary diagnosis of opioid use and did not include patients who presented to the emergency room with an overdose and not admitted for observation. The goals and analysis for this study were descriptive and to examine trends in our hospital of patients with any admission or discharge diagnosis related to substance use disorder. Additional analysis was performed to identify information that may be useful to our clinicians, administration, and the county public health department including changing demographics of this population, other medical issues including types of infections and MRSA frequency, trauma, discharge follow-up, and home address locations as an indicator of geographic areas for admissions. Records were identified using a database query, extracted, and de-identified. Microbial cultures and antibiotic resistance results were extracted from the Department of Clinical Pathology, Microbiology Section, and matched by patient medical record number to the database for the same time period. Case fatality was calculated based on in-hospital mortality of the study population.

### 2.2. University Hospital

University hospital is located in Syracuse, NY, and the major teaching hospital of the State University of New York (SUNY) Upstate Medical University. This is a 735-bed facility and major referral hospital for the area for a higher level of care and includes a catchment area that extends north from the Canadian border, south to the Pennsylvanian border, west to Rochester, and east to Albany.

### 2.3. IRB Approval

This protocol was reviewed and approved with waiver of requirement to obtain informed consent by the Upstate Medical University Institutional Review Board, #1355962-2.

### 2.4. Statistical Methods

Data were analyzed for demographics and temporal and spatial trends with individual parameters examined by chi-square and analysis of variance (ANOVA), as appropriate, using GraphPad Prism version 5.02, GraphPad Software Inc., San Diego, CA, IBM SPSS version 25, Armonk, New York, and R version 3.3.3 published on 2017-03-06. In particular, R packages “zipcode” and “maps” were used for the zip code plot. The statistical analysis of the study was exploratory in nature, and the selection of the analysis results for reporting was subject to the authors' clinical expertise. The *p* values from the study were provided to indicate the probability of the observed data at random, without adjusting the multiplicity issue.

## 3. Results

### 3.1. Number of Hospital Admissions per Year

**Figure 1:**
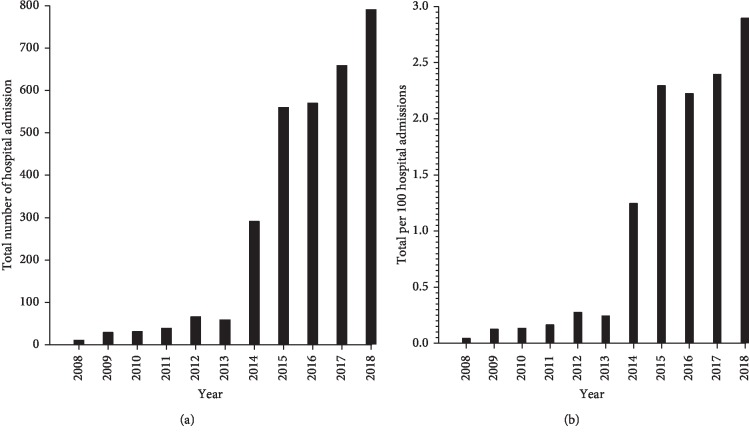
Hospital admissions by year. (a) Total hospital admissions for patients with a opioid use (OU)-related diagnosis by year. (b) Hospital admissions for patients with a opioid use (OU) related diagnosis per total hospital admissions.

The number of hospital admissions with an OU-related admission or discharge diagnosis increased substantially from 2008 to 2018 (Figure 1(a)). In 2008, there were 12 admissions, which increased to 792 admissions in 2018. The total number of hospital admissions for the 11-year period was 3,117. The percent by year of the total OU-related admissions from 2008 to 2018 was 0.4, 1.0, 1.0, 1.3, 2.1, 1.9, 9.4, 18.0, 18.3, 21.2, and 25.4 respectively. The years 2015 to 2018 were associated with 82.9% of all admissions for the 11-year period corresponding to the increase in opioid deaths seen in Central NY (data not shown). To account for changes in total hospital admissions that may bias these results (total hospital admissions have remained constant through the study year), total OU admissions were calculated as a proportion per 100 total hospital admissions per year (Figure 1(b)). In 2008, this proportion was 0.05/100, which increased to a peak of 2.9/100 in 2018, a 58-fold increase in admissions due to OU.

### 3.2. Hospital Discharge and Mortality

**Figure 2:**
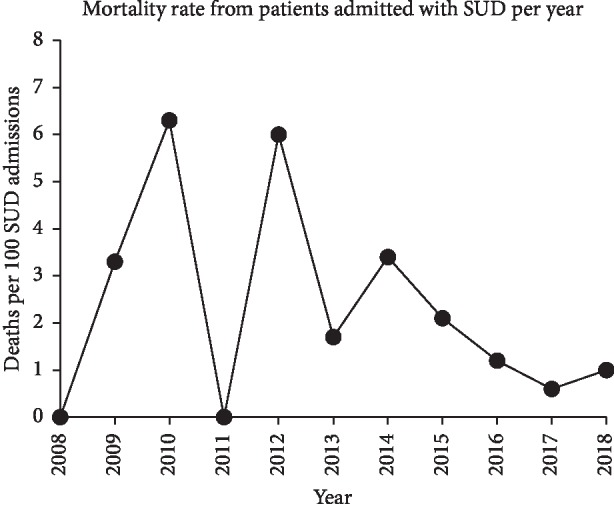
Mortality rate per 100 OU admissions for patients with an opioid use-related diagnosis by year.

Overall, during the 11-year period, there were 49 deaths for a case fatality of 1.2 per 100 OU admissions. Mortality for OU admissions varied by year with a peak of 6.3 and 6.0 per 100 OU admissions in 2010 and 2012, respectively (Figure 2). There was a significant decrease in case fatality between the low-admission years of 2008–2013 (3.3%) as compared with the high-admission years of 2014 to 2018 (1.4%) (*p*=0.0454, chi-square with Yates' correction of 4.0 and Fisher's exact test). Patient disposition on discharge included 415 (13.3%) who left against medical advice; 407 (13%) were discharged to a rehabilitation facility, psychiatric facility, or skilled nursing care facility; and 1,819 (58.4%) were discharged to home or self-care.

### 3.3. Demographic

**Figure 3:**
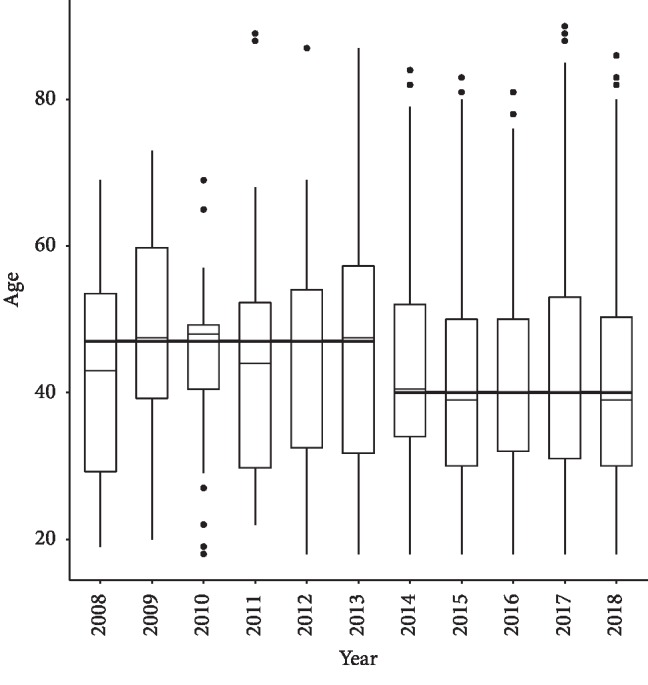
Box-plots of age for the time periods 2008–2013 and 2014–2018 for patients with an opioid use-related diagnosis.

**Figure 4:**
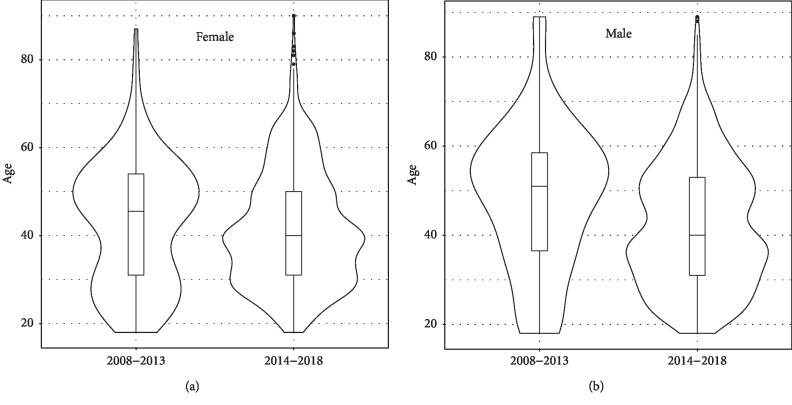
Violin plots of age by gender for the time periods 2008–2013 and 2014–2018 for patients with an opioid use-related diagnosis.

The gender differences between male and females who were admitted with a diagnosis of OU fluctuated from 2008 to 2018. The proportion females in 2008, 2009, 2010, 2011, 2012, 2013, 2014, 2015, 2016, 2017, and 2018 was 50%, 30%, 28%, 45%, 33%, 38%, 51%, 38%, 46%, 43%, and 47%, respectively. For all years, the proportion of females and males was 44% and 56%, respectively. When examining gender differences during 2008 to 2013 as compared with the peak years in 2014 to 2018, the proportion of females increased 8.7% (*p*=0.011 from Pearson chi-square test) from 36.1% during 2008–2013 to 44.8% during 2014–2018. The median age for all years was 40 years (SD of 13.7 years). This did not significantly differ by year (data not shown). Examining age and gender differences between the low-admission years of 2008 to 2013 to the high-admission years of 2014–2018, there were significant differences in age by gender. As shown in the box plot of age by years (Figure 3), yearly median ages are very similar within each time period. Median age drops 7 years (*p*=0.001 from Wilcoxon rank-sum test), from 47 (IQR 32–54) during 2008–2013 to 40 (IQR 31–51) during 2014–2018.  Figure 4 demonstrates that for females, the median age drops 11 years, from 51 (IQR 36–58.5) to 40 (IQR 31–53) years. For males, the median age drops 5 years, from 45 (IQR 31–54) to 40 (IQR 31–50) years. Two-way ANOVA on log-transformed age confirms that patients become younger after 2014 (*p*=0.009), and the age difference is mainly contributed by females (*p*=0.015). Admissions for OU by race primarily were in the white caucasian population, comprising 67.0% of all admissions followed by African Americans (17.6%), American Indians (1.3%), and others in the remainder of race categories. This did not vary by year (data not shown).

### 3.4. Length of Hospitalization

The length of hospitalization from time of admittance to discharge was calculated for each year. Overall, the mean length of stay was 8.55 days (SD 12 days), with a range of 1 to 153 days. The overall mean length of stay by year did not differ statistically with a range of 6 to 9 days (data not shown). When looking at the length of stay for the low-admission years of 2008–2013 to the high-admission years of 2014 to 2018, there was a significant increase in length of stay from a mean of 6 (SD 0.1) days in 2008–2013 as compared with 8.8 (SD 12.2) days in 2014–2018 (*p*=0.001, *T*-test).

### 3.5. Hospital Discharge Diagnosis

Each admission was given one primary discharge diagnosis. The most frequent discharge diagnosis was due to infections (466, 15.0% of discharge diagnosis). Of infections, the most common were cellulitis (176, 37.8%), cutaneous abscess (65, 13.9%), osteomyelitis (56, 12%), pneumonia (46, 9.9%), acute endocarditis (27, 5.8%), intraspinal abscess (23, 4,9%), and sepsis (16, 3.4%). Trauma was the second most common discharge diagnosis (180, 5.8% of discharge diagnosis) with fractures of various bones occurring in 158 (87.8% of trauma-related discharge diagnosis) followed by subdural hematoma (22, 12.2% of trauma-related discharge diagnosis).

### 3.6. Methicillin-Sensitive and -Resistant *Staphylococcus aureus*

We specifically examined the total number and proportion of methicillin-resistant *Staphylococcus aureus* (MRSA) and methicillin-sensitive *Staphylococcus aureus* (MSSA) culture results in the total hospital population, excluding patients admitted with OU and in the OU population. From 2008 to 2018, there were a total of 7,915 culture results positive for *S. aureus*, of which 3,534 (44.6%) were MRSA. The total number of positive cultures did increase every year from 559 in 2008 to 945 in 2018; however, the percentage of MRSA per year did not change significantly from 47.2% in 2008, a peak of 53.5% in 2009, to 45.9% in 2018.  Table 1 demonstrates the total number and rates of MRSA in hospitalized patients admitted with and without a diagnosis of OU. Over the 11-year period in patients admitted without OU, there were 7,029 *S. aureus*-positive cultures, of which 43% were MRSA. This rate did not vary significantly per year. In comparison, there were 886 cultures that grew *S. aureus* from patients with OU during the same time period, of which 58.1% were MRSA (*p* < 0.0001 by chi-square with Yates' correction). The ratio of MRSA rates in the OU as compared with the non-OU population ranged by year from 1.3 to 2.4 and significantly different for every year, except for the years 2008, 2009, 2011, and 2013 (*p* < 0.05 by chi-square with Yates' correction).

### 3.7. Spatial Analysis

**Figure 5:**
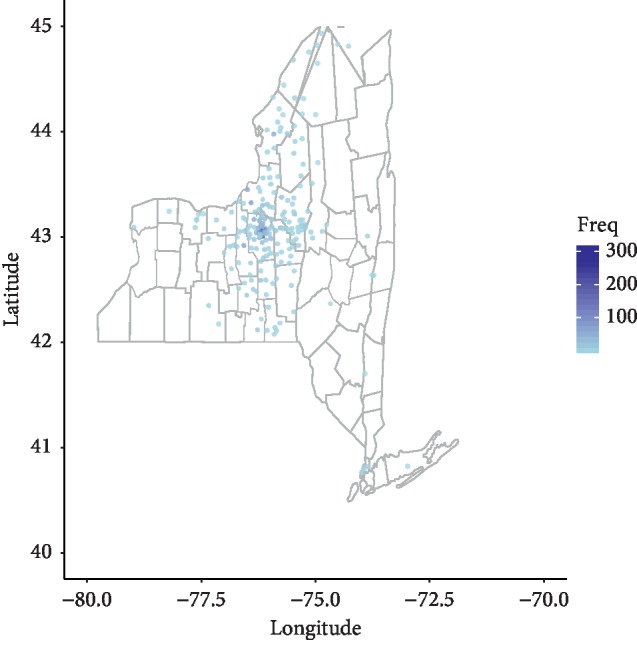
Spatial distribution for patients with an opioid use-related diagnosis OU and hospital admissions 2008 to 2018.

Spatial analysis was performed by zip code and demonstrated in Figure 5.

For all years, the largest number of admissions occurred in zip code 13204, Solvay region, Syracuse, with 299 admissions (10% of total). This was followed by zip codes in a descending number of total admissions, 13202 (213, 6.8%), 13203 (185, 5.9%), 13208 (173, 5.6%), 13205 (167, 5.3%), 13210 (143, 4.6%), and 13207 (105, 3.4%). Collectively, these 7 zip codes had 41% of all hospital admissions for the 11-year period. Figure 3 demonstrates the spatial distribution for all years in Central New York, largely representing the referral area of university hospital with a concentration in and around the city of Syracuse.

## 4. Discussion

Our results reflect the experience of one tertiary-care center in Central New York during the national opioid epidemic to identify local areas of need and interventions to this public health problem. Our findings demonstrated that we have experienced a dramatic increase in the number of hospital admissions of patients with opioid use (OU), specifically related to heroin and intravenous drug use (IVDU). Our experience is largely reflective of the national experience with a large proportion of OU patients admitted with complications of infections related to IVDU [12]. Our findings are surprising with respect to the large number of trauma-related patients with a diagnosis of OU. We demonstrated that the proportion of MRSA isolates in patients with OU are significantly higher than the general hospitalized population reflective of the national experience on the emergence of risk of MRSA in drug users [11]. Spatial analysis of hospital admissions suggested a high concentration of admissions from specific locations within our area, providing an opportunity to target these local communities with education and interventions.

To address this epidemic requires close coordination between local, state, and federal programs focused on identifying individuals with substance use disorder and providing them with the resources to address their addiction. The focus, and appropriately so, has been on opioid-related deaths and funding for Narcan training of first responders and local providers. Hospitalized patients represent a large burden of complications of drug use and an unrealized opportunity to provide resources to address their addiction while hospitalized. In our hospitalized data, the large majority of patients were discharged to self-care. This may be from patient choice, but from our experience, more should be offered to patients to address their addiction. Such efforts are frequently limited because of a lack of funding and lack of emphasis from local and state governments. There should be close coordination with the local public health department to report hospitalizations and to integrate patients into community resources to address their addiction. Spatial analysis of hospital admissions may provide evidence of local communities that could be targeted for education and interventions, especially when resources are limited.

Our analysis identifies areas of improvement in the care of patients with OU and the need for the education of healthcare providers in recognizing and treating addiction. Basic demographic analysis of this population provides important information on the changing demographics of those with OU and valuable information for providers to understand to provide early recognition and treatment for addiction and for administrators to improve resources and addiction services. Addiction services for hospitalized patients, training for medical residents, and addressing barriers for treatment post-acute care have clearly been shown to result in recovery and sobriety for patients suffering from OU [14–17].

The limitations of this study are its retrospective nature and largely numerator-based analyses. The use of proportion of hospital admissions has an inherent bias and may not reflect the true changes in the population, as a population-based rate would. There is an inherent bias during admissions based on the patient location and referrals. For example, one patient may have had multiple hospital admissions that in this analysis considered as separate admissions. Another major limitation is the use of ICD codes to identify patients, as this largely underrepresents the true burden because physicians underreport and often fail to code for opioid use. The change in ICD codes from ICD-9 to ICD-10 may also result in potential bias and an increase in diagnosis of substance use-related diagnoses. Spatial analysis though useful in this analysis has inherent bias, not controlled for in this study by using population-based rates.

We believe an analysis such as this should be considered by every hospital where opioid deaths are occurring. The information gained is valuable for hospital administrators and physicians to plan for the costs and resources needed for the care of this population and to be proactive in addressing this national health crisis.

## Figures and Tables

**Table 1:** Total number and percentage of methicillin-resistant *Staphylococcus aureus* (MRSA)- and methicillin-sensitive *Staphylococcus aureus* (MSSA)-positive cultures in hospitalized patients with and without an opioid use-related diagnosis.

Year	Hospitalized non-opioid use-related diagnosis			Hospitalized with an opioid use-related diagnosis			Ratio SUD to non-SUD
	Total MRSA	Total MSSA	Total (percent MRSA)	Total MRSA	Total MSSA	Total (percent MRSA)	
2008	264	295	559 (47.2%)	0	0	0 (0%)	—
2009	316	276	592 (53.4%)	1	0	1 (100%)	1.9
2010	343	406	749 (46.8%)	10	1	11 (91.0%)	1.9^*∗*^
2011	254	347	601 (42.3%)	2	0	2 (100%)	2.4
2012	302	387	689 (43.8%)	14	0	14 (100%)	2.3^*∗*^
2013	288	374	662 (43.5%)	0	0	0 (0%)	—
2014	224	432	656 (34.1%)	67	28	95 (70.5%)	2.1^*∗*^
2015	236	319	555 (42.5%)	84	70	154 (54.5%)	1.3^*∗*^
2016	219	321	540 (40.6%)	91	77	168 (54.2%)	1.3^*∗*^
2017	277	447	724 (38.3%)	108	90	198 (54.5%)	1.4^*∗*^
2018	296	406	702 (42.2%)	138	105	243 (56.8%)	1.3^*∗*^
Total	3,019	4,010	7,029 (43.0%)	515	371	886 (58.1%)	1.4^*∗*^

^*∗*^Significant by chi-square with Yates' correction, *p* < 0.0.05.

## Data Availability

Data availability is limited due to IRB rules and protected information.

## Supplementary Materials

Supplementary Materials ICD-9 and 10 codes were used to identify patients for this study analysis.
