# Supplementary material for: Determining the Impact of the Opioid Crisis on a Tertiary-Care Hospital in Central New York to Identify Critical Areas of Intervention in the Local Community
Source: J Addict. 2020 Mar 12;2020:3956187. doi: 10.1155/2020/3956187 (PMC7091543; doi:10.1155/2020/3956187)
Supplement: Supplementary Materials — ICD-9 and 10 codes were used to identify patients for this study analysis. [file 3956187.f1.pdf]

Supplemental Table 1. ICD-9 and 10 codes used to identify patients for this study analysis.

304.00–304.03	Opioid type dependence
304.70–304.73	Combinations of opioids with any other

305.50–305.53	Nondependent opioid abuse
965.00	Poisoning by opium
965.01	Poisoning by heroin
965.02	Poisoning by methadone
965.09	Poisoning by other opiates and related narcotics
E850.0	Heroin poisoning
E935.0	Heroin, adverse effects
**Other**	
304.60–304.63	Other, specified drug dependence
304.90–304.93	Unspecified drug dependence
305.90–305.93	Other, mixed or unspecified drug abuse
V654.2	Counseling, substance use

ICD10 Codes	'T40.0X5','T40.0X5A','T40.0X5D','T40.0X5S','T40.0X6','T40.0X6A','T40.0X6D','T40.0X6S','T40.1','T40.1X','T40.1X1','T40.1X1A','T40.1X1D','T40.1X1S','T40.1X2', 'T40.1X2A','T40.1X2D','T40.1X2S','T40.1X3','T40.1X3A','T40.1X3D','T40', 'F11.15','F11.150','F11.151','F11.159','F11.18','F11.181','F11.182','F11.188','F11.19','F11.2','F11.20','F11.21','F11.22','F11.220','F11.221','F11.222', 'F11.229','F11.23','F11.24','F11.25','F11.250','F11.20','F19.20'
